# Improving interference control without conflict exposure: prefrontal fNIRS-decoded neurofeedback training

**DOI:** 10.1117/1.NPh.12.4.045009

**Published:** 2025-12-03

**Authors:** Lingwei Zeng, Wanying Xing, Di Wu, Minghao Dong, Yimeng Yuan, Xiuchao Wang, Zhihong Wen

**Affiliations:** a Fourth Military Medical University, Department of Medical Psychology, Xi’an, China; b Fourth Military Medical University, Department of Aerospace Medicine, Xi’an, China; c Xidian University, School of Life Science and Technology, Xi’an, China

**Keywords:** functional near-infrared spectroscopy, decoded neurofeedback, multivariate pattern analysis, interference control

## Abstract

**Significance:**

Traditional exposure therapy or cognitive training requires repeated presentation of unwanted stimuli, whereas localizationist neuromodulation overlooks individual variation. We propose a closed-loop neuromodulation approach termed functional near-infrared spectroscopy-decoded neurofeedback training, designed to modify prefrontal hemoglobin dynamics and neural activity patterns.

**Aim:**

We aim to enhance interference control without interfering stimuli using a data-driven, individualized, time-resolved decoded neurofeedback, potentially offering a balanced compromise and an alternative to traditional approaches.

**Approach:**

We employed a randomized, double-blind, between-group design. Both the decoded neurofeedback group (DecNef, n=20) and the Sham group (Sham, n=25) developed individualized decoders with a 1-s temporal resolution following the color-word Stroop task (CWST) before training. Both groups received decoded neurofeedback training sessions lasting 25 min daily for three consecutive days, but there was a gap in their decoding accuracy due to differences in sample size. Interference control was assessed via CWST at three timepoints: pre-training (pre-test), post-training (post-test), and 1-week follow-up.

**Results:**

There was no significant difference in feedback scores between groups, but the Stroop effect of reaction time (RT) in the DecNef group showed a significant reduction compared with the Sham group, both at post-test (t=3.056, p=0.004) and follow-up test (t=2.180, p=0.035). The difference wave amplitude (incongruent minus congruent trials) for hemodynamic response functions significantly decreased at post-test in the DecNef group (within a continuous period of 7 to 12 s, p<0.05), but not in the Sham group. Multivariate pattern analysis (MVPA) revealed significantly higher classification accuracy in the DecNef group compared with the Sham group (t=2.370, p=0.024); furthermore, this classification accuracy showed a significant negative correlation with changes in the RT Stroop effect (r=−0.36, p=0.015).

**Conclusions:**

We proposed a closed-loop neuromodulation approach designed to modify prefrontal neural dynamics, with its core innovation lying in time-resolved individualized decoding. This method can significantly improve cognitive function such as interference control while avoiding exposure to unwanted stimuli and has potential for cognitive enhancement and the treatment of psychological disorders such as phobias and post-traumatic stress disorder.

## 1 Introduction

The Stroop effect reflects the ability to maintain the goal and inhibit unwanted thoughts or attention in the face of conflicting stimuli, which serves as a measurement of interference control.^1^^,^^2^ Traditional cognitive training enhances conflict resolution abilities through repetitive practice of relevant tasks or improves brain efficiency using neural modulation tools.^3^^–^^6^ The former relies on the task itself and lacks neural directionality specificity, whereas the latter operates independently of tasks and is theory-driven, ignoring individual and intricate spatiotemporal pattern differences. We propose a data-driven neural regulation method here, by extracting individualized time-resolved brain activity patterns and building a decoder to facilitate closed-loop, real-time self-regulation training, which is termed decoded neurofeedback training.^7^^,^^8^

Interference control, also termed inhibitory control of attention, enables us to selectively attend to the goals stored in working memory (WM) while suppressing attention to other stimuli.^9^ In the color-word Stroop task (CWST), the difference in reaction time (RT) (or accuracy) between congruent and incongruent trials serves as an indicator of conflict resolution ability, also known as inhibitory control—a vital capability for human survival. Deficits in inhibitory control are core symptoms of various psychiatric disorders, including attention-deficit hyperactivity disorder (ADHD), alcohol and substance-use disorders, borderline personality disorder, and neurological disorders, such as Parkinson’s disease.^10^ Traditional neurofeedback training targets specific brain regions activated by inhibitory processes, typically involving subregions of the prefrontal cortex (PFC).^11^^,^^12^ These approaches are based on reductionist thinking and univariate analysis methods. However, some researchers argue that psychological levels cannot claim causal or explanatory priority and that a holistic research strategy is necessary for progress in psychopathology research,^13^ and it is reasonable to develop a personality-oriented personalized psychiatry.^14^ Given that the activated brain regions involved in inhibitory control may be scattered,^15^^,^^16^ task-specific and individualized,^17^ multivariate pattern analysis (MVPA), as an individualized data-driven method, is a reasonable choice.

Unlike univariate analysis, MVPA can capture complex data structures by considering pattern information across multiple variables, such as voxels within the brain or time.^18^ The core idea of MVPA is to decode cognitive states or stimuli by analyzing underlying brain activity through classifiers or regression models. This approach not only reveals the spatial-temporal characteristics of brain activity but also provides higher-level information integration capabilities, aiding researchers in gaining a deeper understanding of neural mechanisms. Currently, MVPA is widely used to decode emotions, cognition, or consciousness states,^19^^–^^23^ it is also used in neurofeedback training and perceptual learning studies. To help humans acquire perceptual knowledge, separate from experience, study, or instruction, Iordan has sculpted new visual categories into the human brain and their impact on individual behavior by functional magnetic resonance imaging (fMRI)-decoded neurofeedback training.^24^ A key challenge across these studies lies in identifying the underlying cortical patterns of cognitive states, where signal modality constitutes a critical consideration.

Functional near-infrared spectroscopy (fNIRS) is a brain imaging modality, which can detect fluctuations of oxyhemoglobin (Oxy-Hb or HbO) and deoxyhemoglobin (Dxy-Hb, or reduced hemoglobin, HbR). Although Oxy-Hb remains the “workhorse” for task-based fNIRS due to its higher amplitude and robustness, Dxy-Hb is “lower sensitivity.” Dxy-Hb is a trade-off for specificity to oxygen metabolism—requiring tailored experimental designs but offering unique biological insights.^25^^,^^26^ For this reason, many univariate analyses in fNIRS studies only examine one type of signal, thereby losing substantial useful information. FNIRS provides moderate temporal and spatial resolution between fMRI and Electroencephalogram (EEG), while also having better ecological validity, making it significantly advantageous for decoded neurofeedback. Combined with MVPA, fNIRS maximizes the use of channel and chromophore information without significantly increasing model complexity. Therefore, fNIRS-decoded neurofeedback may be a potential alternative to fMRI- and EEG-decoded neurofeedback in clinical and neuroscience research.

Neurofeedback training is a form of perceptual learning that allows for unconscious and covert.^3^^,^^27^ Participants may not be aware of what they have learned and do not require any regulation strategies. An fMRI decoded neurofeedback study revealed that participants exhibited perceptual learning for specific visual stimulus features without any strategies to induce specific neural representations, nor did they need to be aware of the content of the reinforced neural representations.^28^ Another related study proposed that fear can be reduced by pairing rewards with the activation patterns of fear in the visual cortex through training, whereas participants remain unaware of the content and purpose of the procedure.^29^ This approach could represent a preliminary step toward new unconscious-processing–based therapies for fear-related disorders such as phobias and post-traumatic stress disorder (PTSD), serving as an alternative to traditional exposure therapy, given that repeated exposure to terrifying stimuli is not always appropriate.

This study proposed a personalized training protocol. MVPA was used to extract the conflict inhibition-related activation patterns for individuals during pre-test CWST. Subsequently, decoded neurofeedback was employed to induce these spatiotemporal patterns in the cortex, which enhanced the targeted cognitive functions without exposing participants to conflict stimuli.

## 2 Materials and Methods

### 2.1 Trial Settings and Sample Size

The trial was conducted in Xi’an, China, from December 2024 to May 2025 and adopted a randomized, double-blind design. The study consisted of cognitive performance measurements at three time points: before the training (pretest), 1 day after the training (posttest), and 1 week after the training (follow-up test).

The sample size was calculated via G*Power version 3.1,^30^ where a mixed-design repeated-measures analysis of variance (ANOVA) model was adapted. We expected a medium inhibitory control performance effect on the basis of a previous neurofeedback study that reported a medium-to-large effect on executive functions.^31^ The effect size was set at 0.25, α=0.05, 1−β=0.8, and the number of measurements was 2. The required minimum sample size was 34 (17 participants in each group).

### 2.2 Participants

This study was approved by the Medical Ethics Committee of the First Affiliated Hospital of the Fourth Military Medical University (Approval No. KY20243578-1). Recruitment advertisements were distributed online and offline in December 2024. Participants were screened via an online questionnaire survey. Inclusion criteria were (1) aged 18 to 25 years, (2) high school education or above, (3) right-handed, and (4) normal or corrected-to-normal vision. Exclusion criteria included (1) history of cranial trauma, heart disease, or cardiovascular diseases; (2) diagnosed psychiatric disorders or history of mental illness; and (3) color blindness or color weakness.

The participants were all medical college students, and subjects who had recently participated in similar experiments were excluded. A total of 48 healthy adults were recruited to participate in this study. All subjects carefully read and signed the informed consent form prior to participation. The participants were randomly assigned to the sham feedback group (Sham) or the fNIRS decoded neurofeedback group (DecNef).^32^ Their gender, age, and educational background were balanced. The group assignments corresponding to participant IDs were pre-defined in the experimental software. The experimental procedure and duration were identical for both groups of participants, differing only in the classification models used. Thus, neither the participants nor the experimenter was aware of the group assignments throughout the experiment. All the subjects completed the Edinburgh inventory,^33^ and all of them were right-handed. The data from three subjects were excluded because of incomplete data records. The data from 25 subjects in the Sham group (11 females) and 20 subjects in the DecNef group (10 females) were used in this study. Independent t-tests showed no significant differences in age or education between groups (Table 1).

**Table 1:** Demographic information for the participants in each group.

	Number of participants	Age (SD) in years	Education years (SD)
Sham	25 (11 females)	20.88 (1.72)	14.75 (1.76)
DecNef	20 (10 females)	21.70 (1.49)	15.57 (1.63)
p Value	—	0.10	0.12

### 2.3 Brain Imaging Tools and Channel Layout

**Fig. 1:**
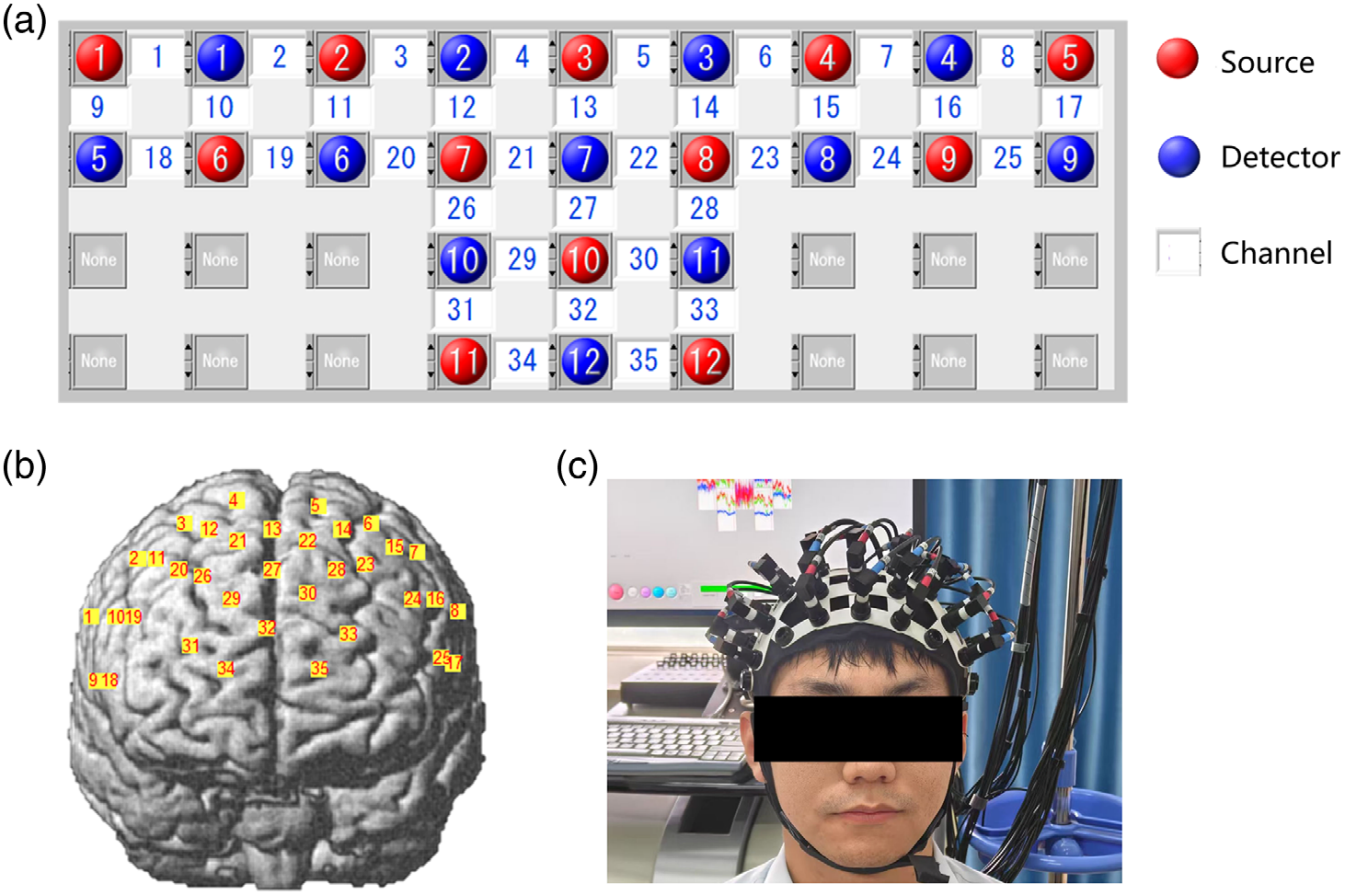
Channel layout schematic. (a) Source–detector array on the LABNIRS system (red discs: sources; blue discs: detectors; white squares: measurement channels). (b) Channel locations on a head model (NIRS-SPM). (c) A subject wearing the fNIRS fiber holder.

In this study, an optical brain function imaging device (LABNIRS, Shimadzu Corp., Japan) was used to monitor the concentration of hemoglobin via a three-wavelength near-infrared semiconductor laser (780, 805, and 830 nm). We designed a “T” layout on the frontal lobe, and the probe arrays allowed for 35 different measurement channels, with 3.0 cm of source-detector separation. Note that no short channel was employed in our experiment, as shown in Fig. 1.

The 3D spatial coordinates, measured relative to a standard head model via the 3D locator (Fastrak3d, Polhemus Corp., USA), were converted into MNI space and mapped to Brodmann parcellations via probabilistic registration using NIRS-SPM.^34^ The results indicate that these channels encompassed most of the PFC as conventionally defined and described in Carlen’s study,^35^ as shown in Table 2.

**Table 2:** Anatomical labels of channels.

Brodmann area	Right hemisphere	Left hemisphere
9(46)-Dorsolateral prefrontal cortex	1, 10, 18, 19, 29	16, 24, (25), 30
6-Premotor and supplementary motor cortex	3, 4, 12, 13	5, 6, 7, 8, 14
8-Includes frontal eye fields	2, 11, 20, 21, 26, 27	15, 22, 23, 28
45-Pars triangularis Broca’s area	9	17
10-Frontopolar area	31, 32, 34	33, 35

### 2.4 Experimental Protocols

**Fig. 2:**
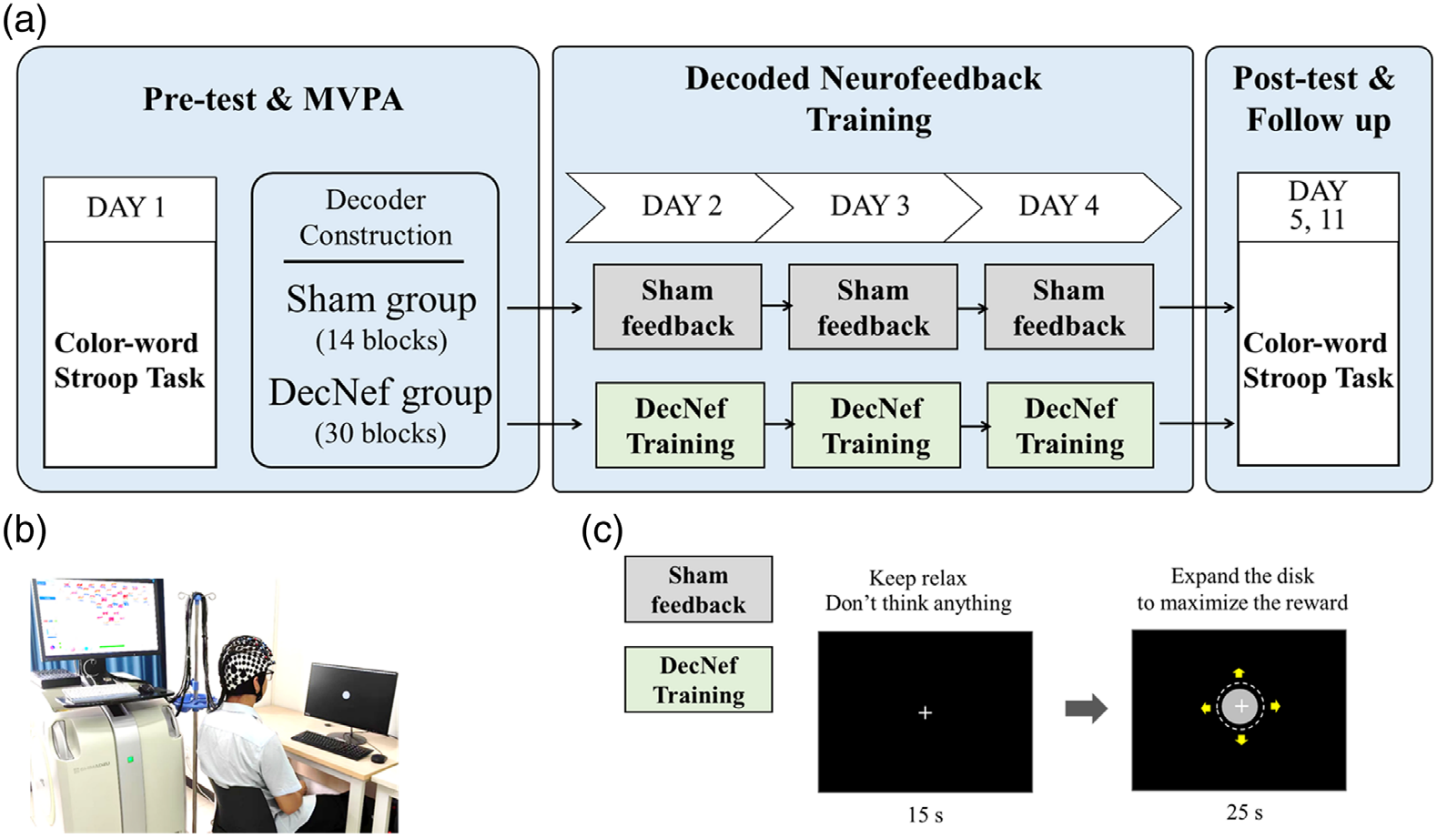
Experimental protocols. (a) Main process of the experiment. (b) Participants and experimental settings. (c) Single block timing for training session.

Each participant completed six sessions within 11 days, including behavioral tests on days 1, 5, 11, and 3 training sessions from days 2 to 4 [Fig. 2(a)].

During the decoder construction period (pre-test and MVPA), participants performed a CWST consisting of 60 blocks (see Sec. 2.5), with all fNIRS data recorded. Subsequently, for the DecNef group, all 60 blocks (30 blocks per condition: congruent and incongruent) were used for feature selection and decoder construction. For the Sham group, however, only the first 14 blocks for each condition (a total of 28 blocks) were reserved. The reduced sample size resulted in lower classification accuracy (closer to the 50% chance level) compared with the full-sample group without creating a significant divergence in feedback scores, thus serving as the Sham control (results were shown in Secs. 3.1 and 3.4).

During the neurofeedback training sessions, there were 30 training blocks per day with a 1-min rest at the end of every fifth block. The participants sat in front of a computer with their eyes 60 to 70 cm away from the screen and kept their bodies, especially their heads, relaxed without moving, as shown in Fig. 2(b). Neurofeedback training comprised two sequential steps. In step 1, participants were instructed to remain relaxed and avoid active thinking for 15 s to establish a baseline. In step 2, participants attempted to maximize a gray disk [Fig. 2(c)] by mental effort for 25 s. The size of the disk indicates the accumulation of probabilities of cortical signals being identified as inconsistent patterns (with a refresh rate of 10 Hz; for the calculation method, see the following Sec. 2.7). The participants were instructed to expand the disk as much as possible; they could use any psychological strategy other than breathing, physical, or facial adjustments; and they were told that a monetary reward (between CNY 300 and 500) would be paid in proportion to the score after the end of all experiments.

### 2.5 Behavioral Testing

**Fig. 3:**
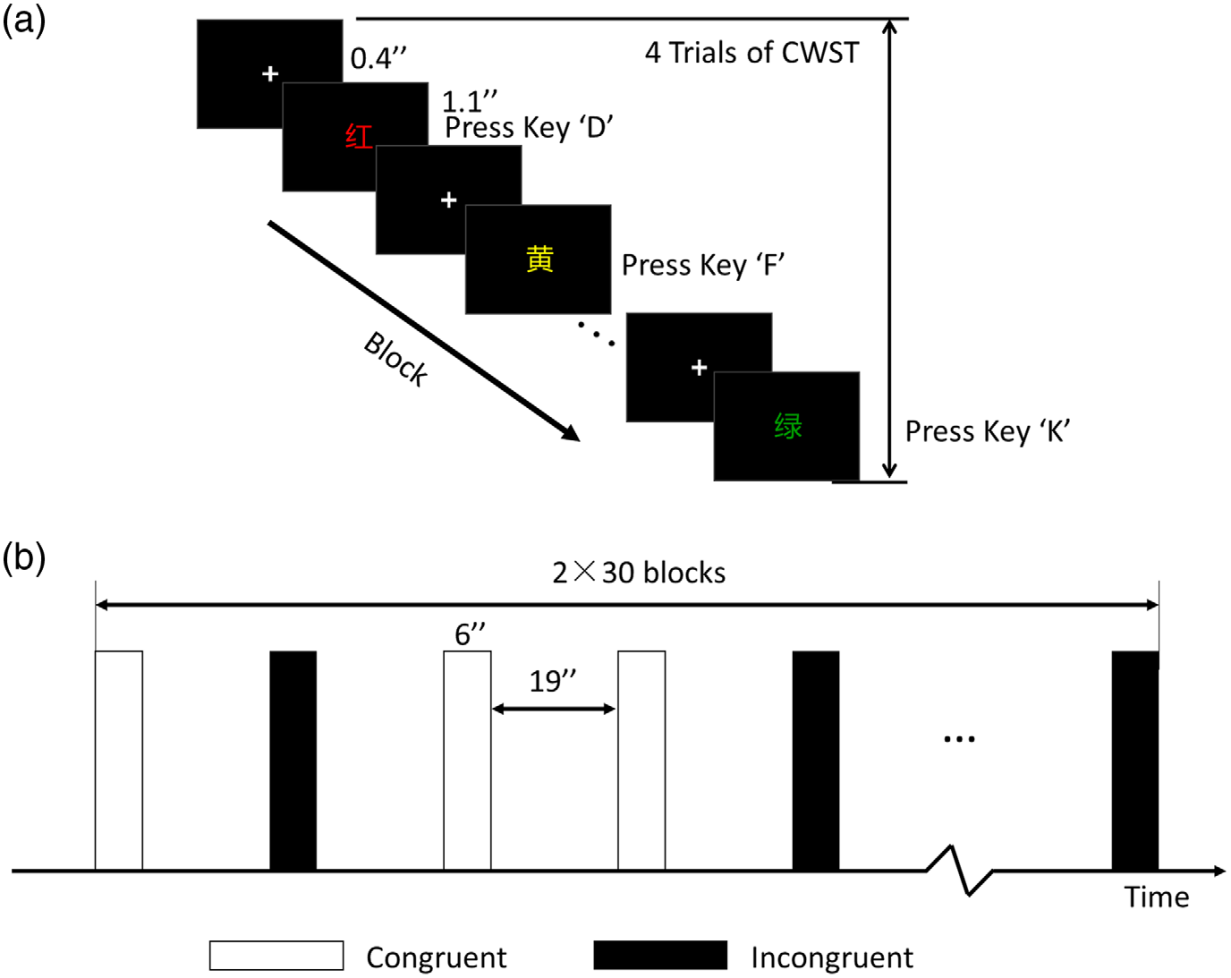
Behavioral test of the CWST. (a) One block of the CWST. (b) Block design and arrangement. Two conditions were included, and each condition consisted of 30 blocks with a counterbalanced order. “+” indicates fixation.

The Chinese version of CWST (four colored Chinese characters) was used in this study as the behavioral test during the pretest, posttest, and follow-up test. During the experiment, each block consisted of four consecutive trials, which were exclusively either congruent or incongruent. A fixation was presented for 0.4 s, and each color-word trial was presented for 1.1 s. The participants rested for 19 s at the end of each block. Participants were instructed to respond to the ink color of the presented words using the corresponding keys: red (D), yellow (F), blue (J), and green (K). A single task session, lasting ∼25  min, was composed of 60 blocks (30 congruent and 30 incongruent) presented in a pseudo-randomized sequence, as shown in Fig. 3.

### 2.6 MVPA and Decoder Construction

**Fig. 4:**
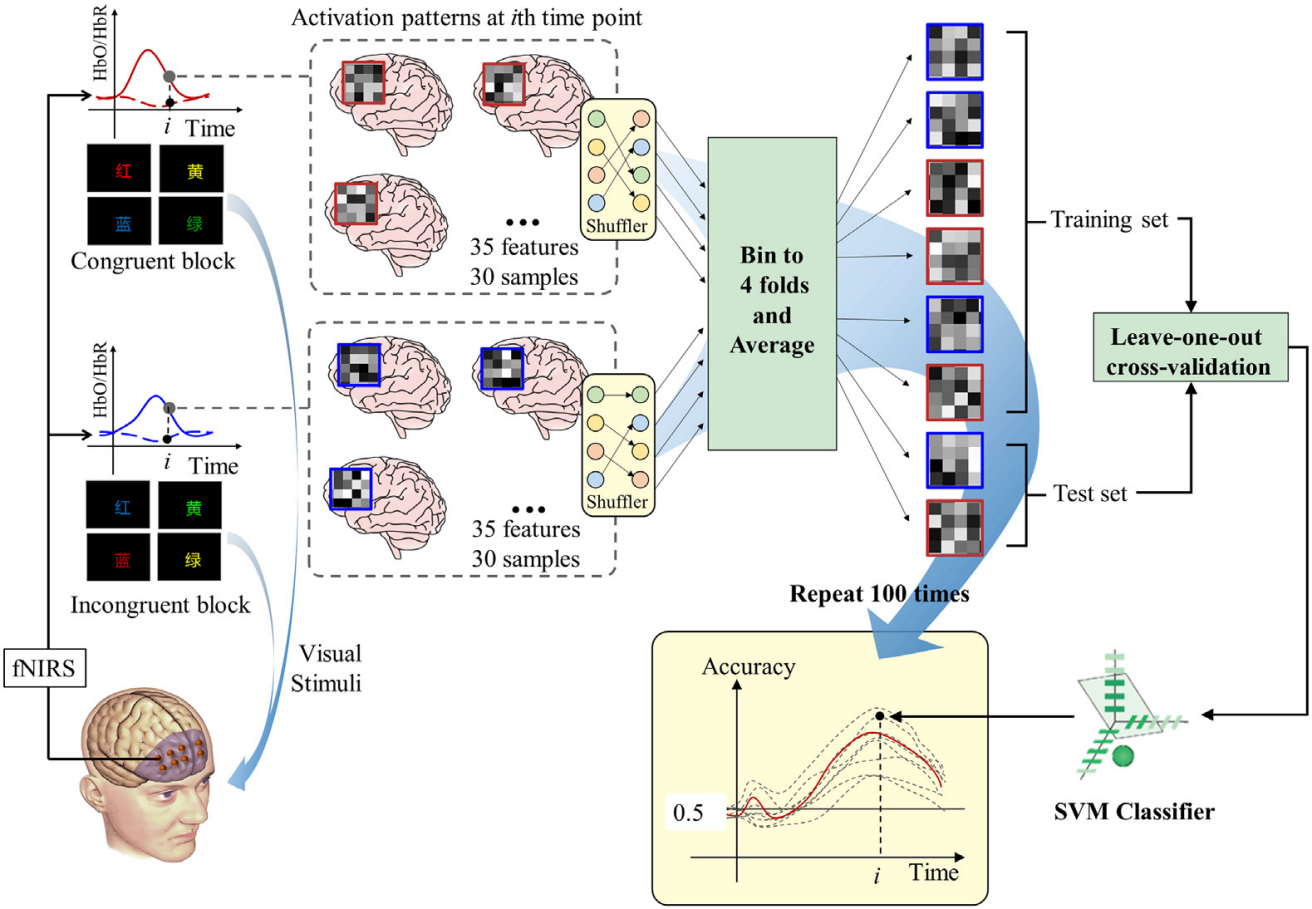
Steps of MVPA. A series of visual stimuli, consisting of congruent and incongruent conditions, was presented to participants. Time-resolved decoding accuracy for each subject was derived by SVM classification of the cortical hemodynamic responses evoked by the two stimulus types. This classification procedure was repeated 100 times with a different random reshuffling of all trials. The resultant decoding accuracies were then averaged to produce the mean classification curve (shown in red solid line).

To test the classification ability of fNIRS data and optimize decoder parameters, Ashton’s MVPA method was executed via MATLAB R2017a (MathWorks Inc., Natick, Massachusetts, USA).^36^ Preprocessed pretest CWST trials were acquired for each subject, following the procedures detailed in Sec. 2.8. There were 30 congruent blocks and 30 incongruent blocks per subject for the DecNef group. For the Sham group, however, only the first 14 congruent blocks and 14 incongruent blocks were used. A total of 140 features comprising Oxy-Hb, Dxy-Hb, and their first derivatives ($\mathrm{Oxy}\text{-}{\mathrm{Hb}}^{\prime }$, $\mathrm{Dxy}\text{-}{\mathrm{Hb}}^{\prime }$) were initially included. The sampling rate was 25.6 Hz, and the step size of MVPA is 1 s without overlap. The algorithm is as follows and shown in Fig. 4.

(1) For one time point i, all congruent and incongruent patterns were individually shuffled (internally randomized via permutation).
(2) All congruent patterns and all incongruent patterns were separately divided into 4 bins. The patterns within each bin were averaged, resulting in 8 averaged patterns (4 congruent + 4 incongruent).
(3) Using a leave-one-out cross-validation, one pair of averaged patterns (one congruent, one incongruent) was held out as the test set, whereas the remaining three pairs were used as the training set. Classification was performed using an SVM with a linear kernel, and the classification accuracy was calculated.
(4) Step 1 (shuffling) was repeated 100 times for each subject’s patterns. The classification accuracy was computed for each shuffle, and these values were then averaged to obtain the mean classification accuracy at the i’th time point.
(5) Steps 1 to 4 were performed for all time points within the time window (−5  s to 25 s relative to stimulus onset), yielding the time-resolved mean classification accuracy for each subject.

MVPA not only investigated the feasibility of fNIRS decoding but also played a crucial role in optimizing decoder parameters. To improve the decoder’s generalization classification accuracy and robustness, feature screening was performed to reserve 35 features with the highest classification accuracy empirically. The 10 to 17 s post-stimulus window was selected for feature ranking while maintaining a consistent feature space throughout the entire decoding period for each subject. However, it should be noted that the individualized decoder changes every second to adapt to the time-varying system. These individualized classification models and feature numbers are stored on the hard drive for subsequent use.

### 2.7 Feedback Scores Calculation

During neurofeedback training, participants performed the task while their personalized decoder was applied in real time to decode brain states. Feedback scores derived from this decoding were presented on a screen to guide participants’ self-regulation. The detailed computation steps were as follows:

(1) During baseline recording, fNIRS data matrices (35 channels × 15 s temporal samples) were obtained for Oxy-Hb and Dxy-Hb concentrations. The mean ($\overset{¯}{Y}$) and standard deviation (SD) were subsequently computed for each chromophore’s time series independently.
(2) During the regulation period, data within the 1-s window preceding i’th time point were extracted at 0.1-s intervals (10 Hz). Only the 35 pre-selected features were reserved as input, denoted as $Y_{i}$.
(3) $Y_{i}$ was normalized to z-score using the following formula.
  $$Z_{i}=\frac{Y_{i}-\overset{¯}{Y}}{SD}.$$
(4) The probability of being classified as incongruent stimulus is as follows:
  $${\text{Score}}_{i}={M\text{odel}}_{i}(Z_{i})\;\;(0\leq {\text{Score}}_{i}\leq 1).$$
  where ${\text{Model}}_{i}$ indicates the SVM decoder at ith time point.
(5) The gray disk on the screen shows the feedback score and requires the participant to improve the feedback score as much as possible. Repeat steps (1) to (4) for calculating the feedback score until the end of the block.

### 2.8 Preprocessing and Statistics of Offline Data

We performed the following preprocessing steps on offline data via NIRS_KIT v3.1^37^ and Homer 2^25^ based on MATLAB 2017a.

(1) The intensity data (raw data) were converted to optical density (OD) values.
(2) A bandpass filter (cutoff frequency: 0.01 to 1 Hz) was applied to remove linear trends and artifacts preliminarily.
(3) Motion artifacts were identified based on amplitude and standard deviation thresholds. If the signal for any channel changes by more than 60 times of standard deviation or 6 times of amplitude over 3 s interval, then this time point is marked as a motion artifact.
(4) Cubic spline correction was performed on the motion artifacts identified in step 2.
(5) A bandpass filter (cutoff frequency: 0.01 to 0.2 Hz) was applied to the data to further reduce the noise.^38^
(6) The OD data were converted to concentrations. As young adult participants were included in our experiment, we chose [6.0 6.0 6.0] as the differential path length factor (DPF), as suggested in Chiarelli’s study.^39^

If the data were from the task state (Stroop), two additional steps of processing were required.

(7) The denoised concentrations were segmented into blocks and averaged to the hemodynamic response function (HRF).

To investigate whether the DecNef group presented improved cognitive performance compared with the Sham group, we analyzed the Stroop effect across 3 testing stages via mixed-design repeated-measures ANOVA in IBM SPSS Statistics for Windows, version 23 (IBM Corp., Armonk, N.Y., USA). Second, the changes in HRFs before and after training were tested using a point-by-point paired t-test. In addition, the time-resolved classification accuracy in each group was evaluated by MVPA and compared, and the relationship between classification accuracy and cognitive performance was investigated by Pearson correlation analysis. Finally, we conducted a further comparison in classification accuracy among various chromophores and feature sets, offering insights for practical applications. In these analyses, p<0.05 was considered significant.

## 3 Results

### 3.1 Changes in the Feedback Score

**Fig. 5:**
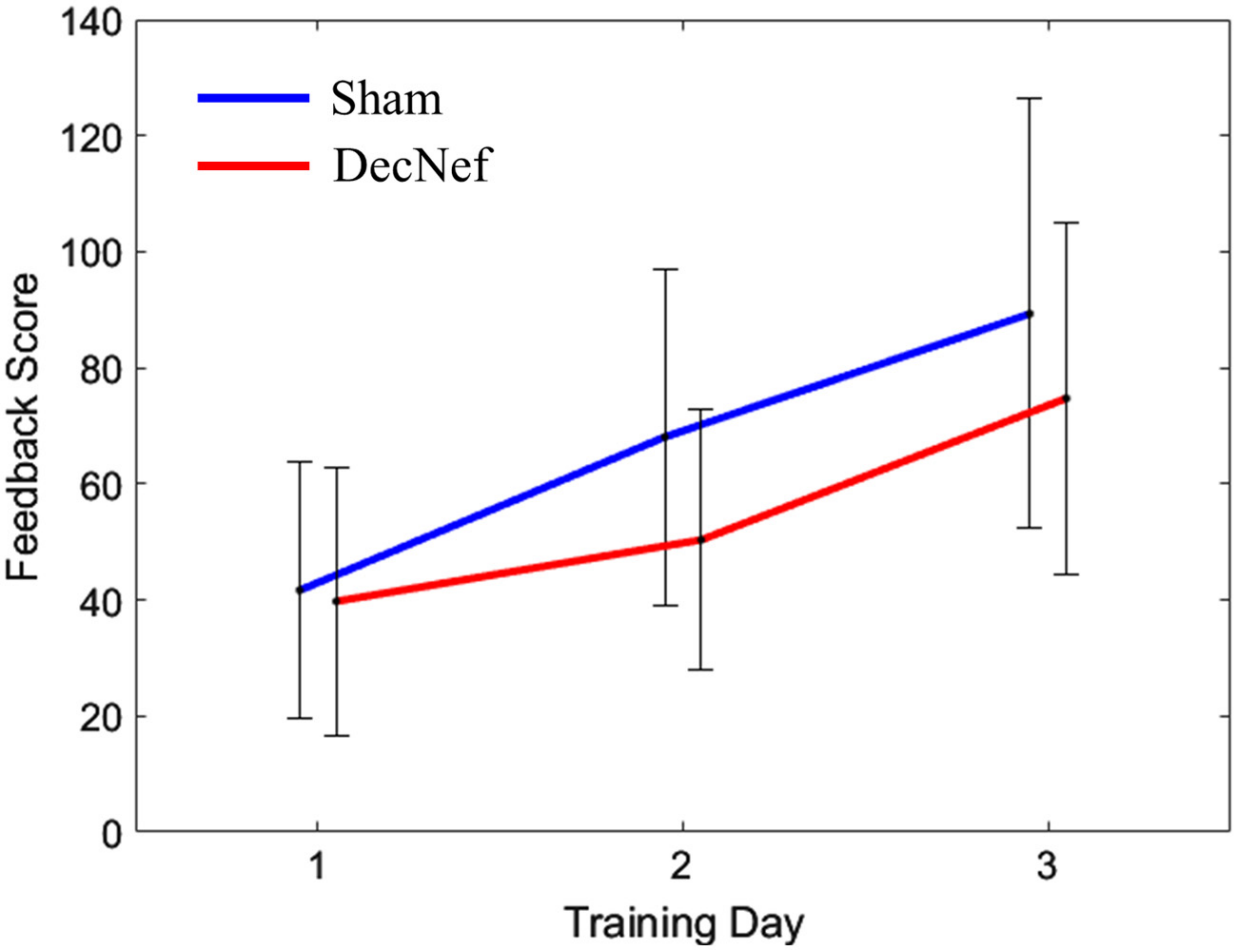
Feedback score of both groups over 3 training days. The error bars indicate the SEM.

Feedback scores of the Sham group and the DecNef group were recorded. The average scores for the Sham group across 3 training sessions were 41±22 (SEM), 68±29, and 89±37, whereas the average feedback scores for the DecNef group were 39±23, 50±23, and 74±30, as shown in Fig. 5. The average scores of both groups increased, but no significant differences were found within or between the groups.

### 3.2 Changes in Inference Control

**Fig. 6:**
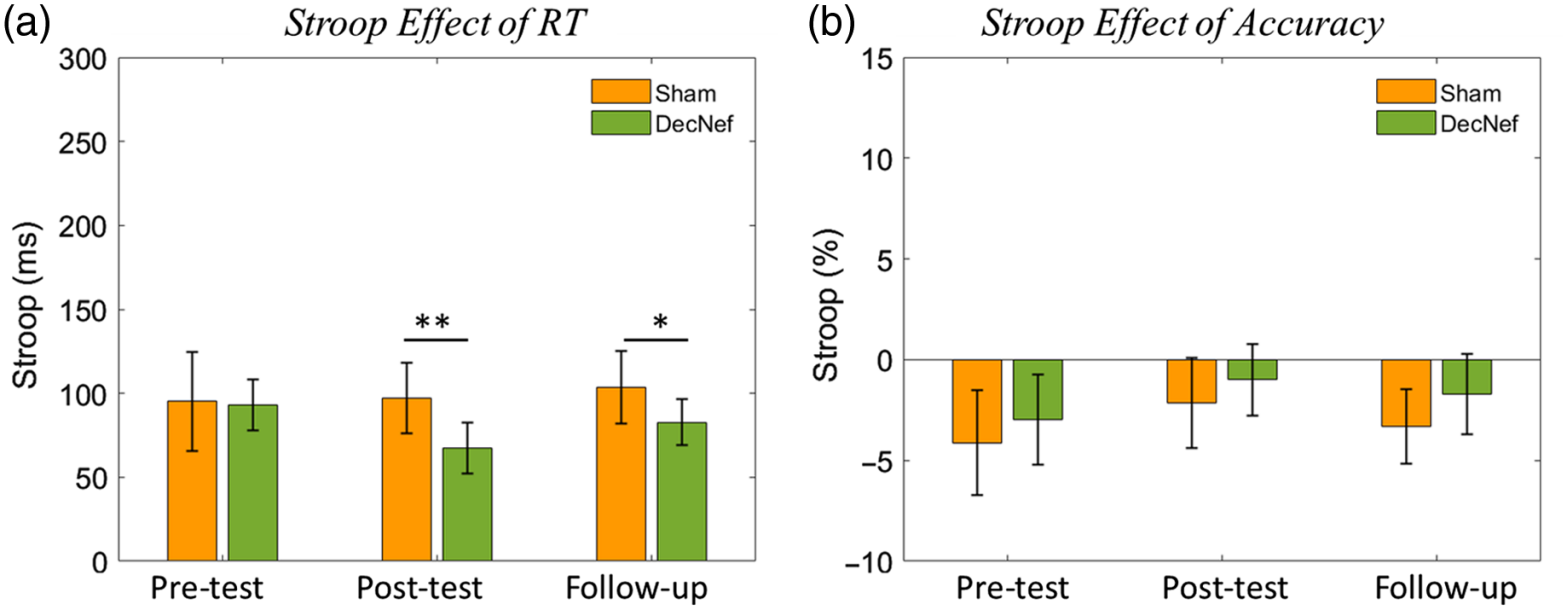
Stroop effect at pre-test, post-test, and follow-up. The left subplot shows the Stroop effect in RT, whereas the right subplot shows the Stroop effect in accuracy. The error bars indicate the SEM for each group. Paired t-test, *p<0.05, **p<0.01.

The Stroop effect extracted at pre-tests, post-tests, and follow-up was analyzed to investigate the immediate and lasting effects of training, which were submitted to a mixed-design repeated-measures ANOVA. We compared the test results between pre-test and post-test, that is, the immediate effect. There was no significant main effect of day and group. The interaction between group and time was significant [F(1,43)=6.250, p=0.016, partial $\eta^{2}=0.127$]. Simple effects were analyzed using a paired t-test. The Stroop effect was not statistically significantly different in the DecNef group (M=93.11, SEM = 15.12) compared with the Sham group (M=95.27, SEM = 29.40) at the pre-test (t=0.173, p=0.864). However, the Stroop effect was statistically significantly lower in the DecNef group (M=67.37, SEM = 15.26) compared with the Sham group (M=97.04, SEM = 21.01) at post-test (t=3.056, p=0.004). We then examined the long-lasting effects of training during follow-up using a paired t-test. The results showed that the Stroop effect of RT in the DecNef group (M=82.78, SEM = 13.71) was significantly lower than that in the Sham group (M=103.68, SEM = 21.46; t=2.180, p=0.035). These results were shown in Fig. 6(a). What is more, the Stroop effects of accuracy were also extracted and submitted to a mixed-design repeated-measures ANOVA. Neither the main effect nor the interaction effect was observed, as shown in Fig. 6(b).

### 3.3 Changes in HRFs of the Stroop Task

**Fig. 7:**
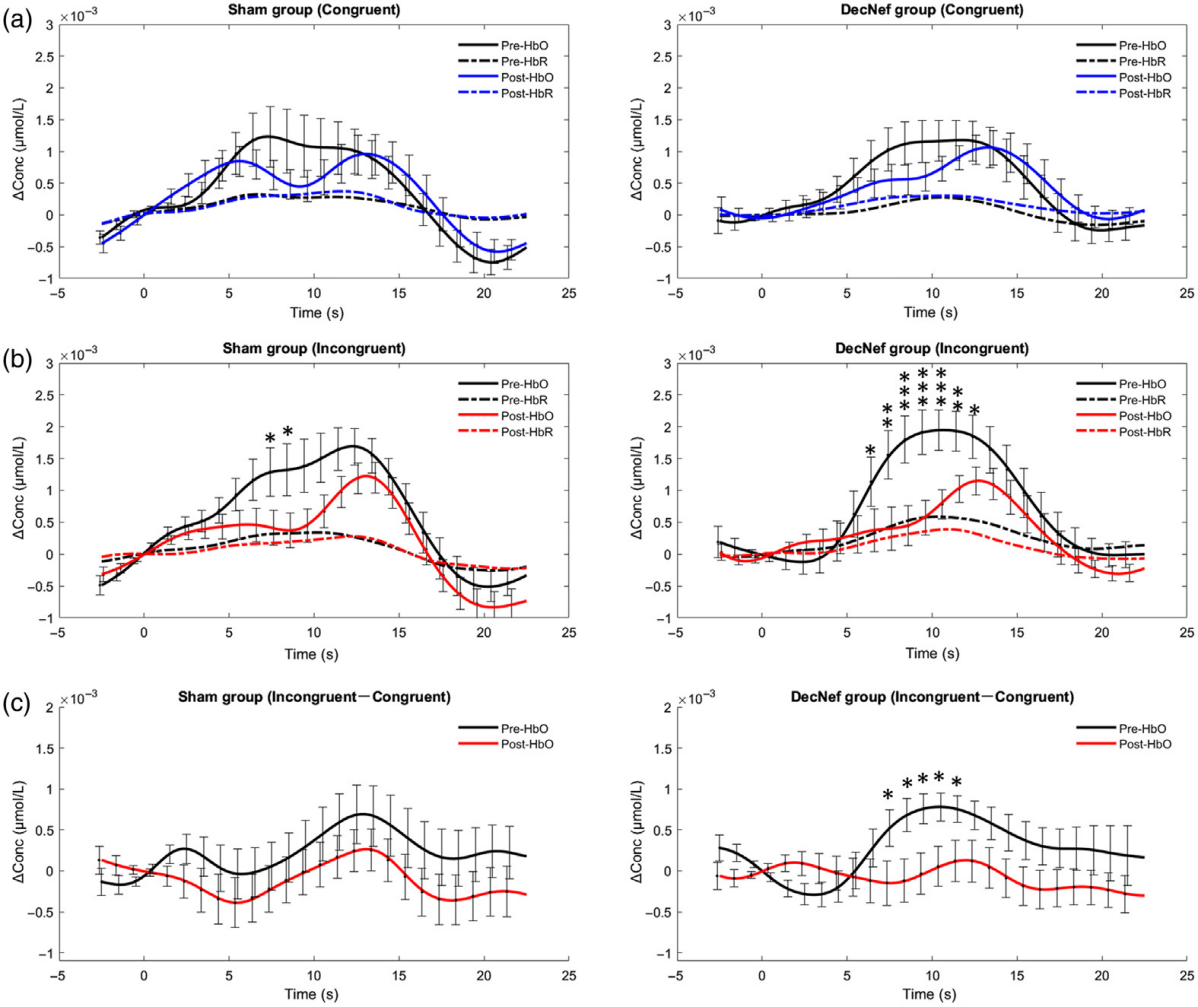
Changes in HRFs and difference waves for the Stroop task before and after training. (a) Grand average HRFs for congruent conditions, which were derived from all channels and all subjects within each group. (b) Grand average HRFs for incongruent conditions within each group. (c) Grand average HRFs for the difference waves, which were calculated by subtracting congruent Oxy-Hb from incongruent Oxy-Hb. Error bars indicate SEMs. Paired t-test, *p<0.05, **p<0.01, and ***p<0.001, uncorrected.

The HRF refers to the process whereby localized neural activity leads to delayed changes in regional cerebral blood flow and blood oxygen levels.^40^ Given the heterogeneity of individual channel selection in decoding models, we averaged the HRFs of all channels. HRFs were extracted and compared before and after training with investigate the training-induced inter-group neural activity differences. We conducted intra-group comparisons of HRFs for conditions of congruent, incongruent, and differential waves, employing point-by-point paired t-tests. Significant differences in within-group pre-post comparisons were observed for both groups, but only for incongruent conditions rather than congruent conditions. Specifically, the Sham group showed significantly greater pre-test Oxy-Hb than post-test Oxy-Hb in the continuous interval of 8 to 9 s (p<0.05); the DecNef group also demonstrated significantly greater pre-test Oxy-Hb than post-test Oxy-Hb in the continuous interval of 7 to 13 s (p<0.05), as shown in Figs. 7(a)–7(b).

Difference waves associated with congruent versus incongruent conditions were extracted to reflect the specific neural activity related to interference inhibition. The results indicated that the Oxy-Hb difference wave was significantly smaller after training compared with before, specifically for the DecNef group, but not for the Sham group. In detail, the DecNef group demonstrated significantly lower difference waves in the post-test compared with the pre-test within a continuous interval of 7 to 12 s (p<0.05), as shown in Fig. 7(c).

### 3.4 MVPA and Feature Comparison

**Fig. 8:**
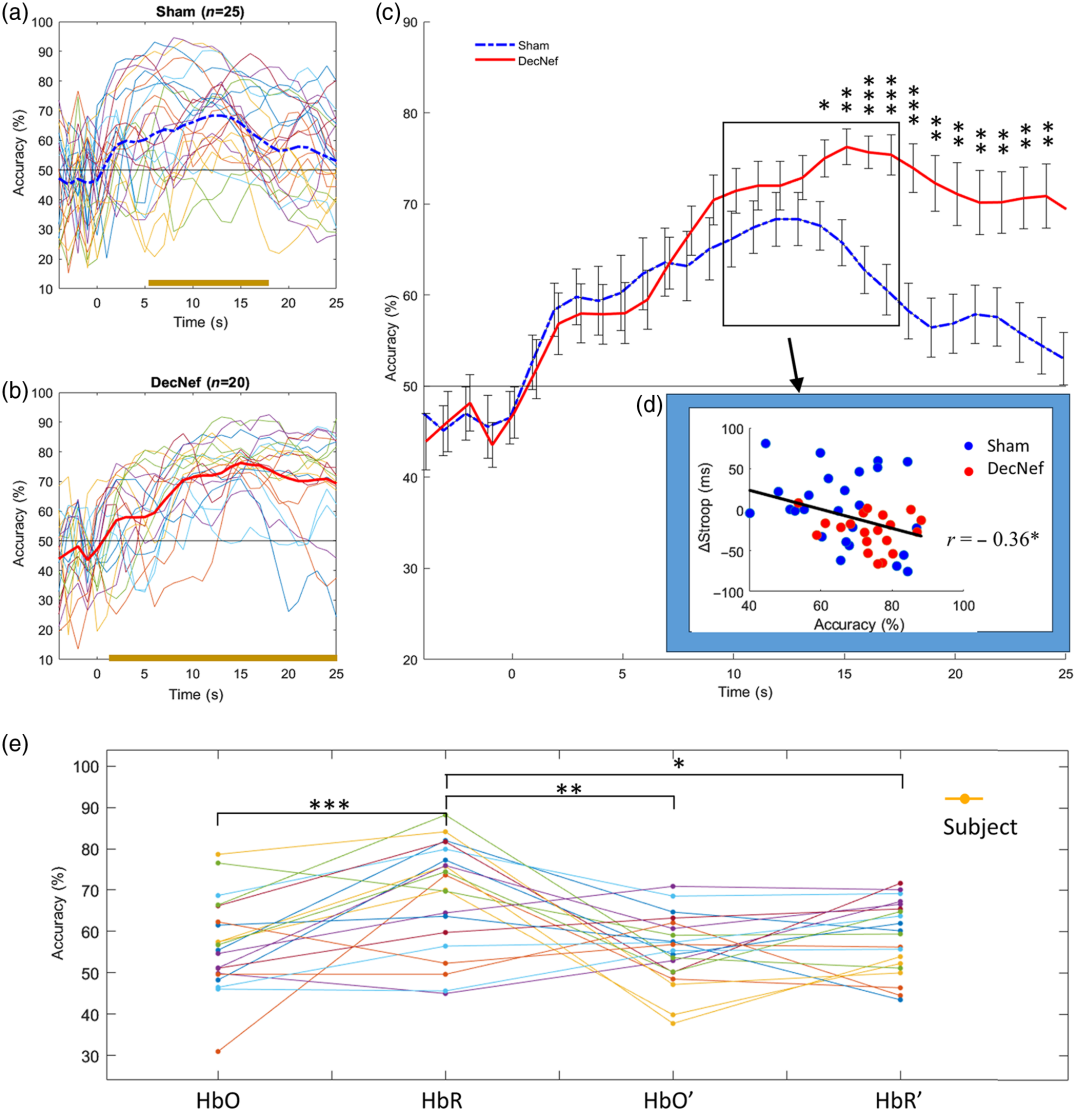
Classifier performance and its correlation with the Stroop effect. (a), (b) Time-resolved classification accuracy for all subjects in the Sham group (blue dashed line) and DecNef group (red solid line). The yellow horizontal line indicates the continuous interval where the classification accuracy is greater than 50%. Zero-test, p<0.05. (c) Average classification accuracy curves for the Sham group (blue dashed line) and DecNef group (red solid line), with error bars indicating SEM. (d) Pearson correlation analysis between the Stroop effect and classification performance, where classification accuracies are derived from the average of the curves between 10 and 17 s. Blue dots indicate the Sham group, and red dots indicate the DecNef group in the scatter plot. (e) Variation in classification accuracy when using different chromophores (Oxy-Hb, Dxy-Hb, and their first derivatives) as Features. Paired t-test, *p<0.05, **p<0.01, and ***p<0.001, uncorrected.

The average classification accuracy of each group was calculated by MVPA and compared using a point-by-point paired t-test. Classification accuracy was higher in DecNef at 14 s post-stimulus (p<0.05), as shown in Figs. 8(a)–8(c). Mean accuracy (10 to 17 s) negatively correlated with Stroop effect (r=−0.36, p=0.015), suggesting that the higher the model’s classification accuracy, the more the interference control is improved, as shown in Fig. 8(d).

A one-way repeated-measures ANOVA was conducted to test the classification capability of different chromophores (and their first derivatives) used as features. This analysis showed a significant main effect of chromophore type on classification accuracy (F(3,19)=6.98, p<0.001). Subsequent post hoc analyses demonstrated that Dxy-Hb outperformed other features in classification (versus Oxy-Hb: t=3.91, p<0.001; $\mathrm{Oxy}\text{-}{\mathrm{Hb}}^{\prime }$: t=3.40, p=0.003; $\mathrm{Dxy}\text{-}{\mathrm{Hb}}^{\prime }$: t=2.83, p=0.011), as shown in Fig. 8(e).

## 4 Discussion

Following the completion of all sessions, interviews were conducted with all participants, covering their awareness of group assignment and the mental strategies they used during training. Neither the participants nor the experimenter was aware of group assignments. Furthermore, the similar and non-significantly different scores between groups suggest an absence of heterogeneity in confidence. Given the double-blind, sham-controlled design, placebo and exercise effects were controlled for. The interview also revealed that keeping attention and imagining new rather than repeated things contributed to their scores, which coincides with the operational definition of interference control.

One issue is that the research design is based on an implicit fundamental assumption: a smaller sample size leads to poorer classification performance. This assumption is supported by several machine learning studies, which indicate that a smaller sample size increases the variability in model estimation and the risk of overfitting.^41^ More critically for this study, an excessively small sample size may fail to adequately represent the feature space of the data, resulting in poorer generalization ability.^42^ Consistent with the hypotheses, the MVPA results revealed the limitations of a small sample size: the Sham group demonstrated classification accuracy closer to chance level. This difference in model performance may account for the observed disparity in behavioral outcomes.

This study demonstrates improved interference control through fNIRS-decoded neurofeedback training for the first time to our knowledge. The results also demonstrated that just three sessions of 25-min individualized decoded feedback training can effectively improve cognitive performance related to conflict resolution without the exposure of conflicting stimuli, with the effects lasting for a week or longer. Although achieving comparable training efficacy to the authors’ earlier targeted brain network neurofeedback study,^43^ this approach required fewer sessions, demonstrating the superior efficiency of decoded neurofeedback. Moreover, the analysis of HRFs revealed a significant decrease in Oxy-Hb during the post-test, indicating an improvement in neural efficiency induced by training. More importantly, the difference wave, which directly reflects the time-varying nature of the conflict process, significantly decreases in the DecNef group rather than the Sham group, suggesting a decrease in oxygen metabolic demand related to interference control. These results demonstrated that decoded neurofeedback training targeting conflict processing specifically engages the conflict mechanism itself, thereby avoiding interference from irrelevant cognitive processes inherent in traditional cognitive behavioral training. This approach exhibits superior cognitive process targeting specificity.

Correlation analyses revealed that classification performance may directly influence training efficacy, where higher classification accuracy is beneficial for enhancing the cognitive ability induced by training. High generalization classification accuracy requires feature screening. MVPA offers a principled method for parameter assessment, resulting in the empirical selection of 35 features with maximal classification accuracy. Furthermore, the optimal time window was identified as 10 to 17 s post-stimulus onset, which corresponds closely with the slow hemodynamic response reported in other studies.^44^^,^^45^

We independently investigated the classification performance of both chromophores and their first derivatives (signal change rates, potentially advantageous if hemodynamic responses do not return to baseline), yielding valuable insights. First, Dxy-Hb showed better classification accuracy, although its signal was noisier and smaller in amplitude than Oxy-Hb suggested by other studies.^46^^–^^48^ Second, derivatives contribute to the overall classification accuracy, despite their individual classification accuracies not being significantly higher than chance. Conventional univariate analyses in fNIRS research often neglect these important yet subtle effects owing to methodological oversimplification. The failure of univariate models to detect these effects highlights MVPA’s critical advantage: leveraging population coding principles rather than isolated voxel responses.

Compared with other theory-driven approaches,^49^^–^^51^ data-driven methods enable the construction of personalized decoders that cater to individuals,^51^^,^^52^ thereby achieving precise and long-lasting neural shaping. This study aimed to target interference inhibition; however, the proposed method is also applicable to other cognitive functions and neural activities, owing to its data-driven nature. Therefore, the finding holds promising application prospects. For instance, for individuals suffering from phobias, a more acceptable treatment alternative to exposure therapy is using decoded feedback training to induce activation patterns associated with fear memories in the brain without directly presenting the feared objects. A similar study based on fMRI-decoded neurofeedback training has been reported.^29^

In neuromodulation, cognitive training relies on task repetition but lacks neuroanatomical specificity, whereas localizationist approaches require intervention targets with explicit cross-subject consistency—a condition fundamentally challenging to satisfy for complex tasks. Our method may be a compromise between the two, offering neural targeting without requiring consistent targets. However, a potentially controversial issue is whether the altered brain and cognitive function involved in this study represent interference control. Given that there is controversy over the neural localization of interference control and inhibitory control,^29^ we did not entangle in the abyss of complex theories, but instead focused on improving behavioral performance and clinical application prospects. This decoding approach selectively excludes non-target neural activations associated with conventional cognitive training paradigms, thereby focusing neuromodulation precisely on the intended psychological processes. A caveat is the task-specificity of training effects. Although Stroop task performance improved significantly, we observed no transfer to the Stop-Signal Task (SST), another inhibitory control paradigm. This suggests that the predictive models are highly task-dependent, relying on specific activation patterns that may not generalize to other tasks even if they have similar cognitive processes, highlighting the functional heterogeneity between tasks.

Some limitations of the study need to be addressed. First, the universal neural targets for enhancing interference control via neurofeedback training remain ambiguous. Our whole-brain analysis using a general linear model revealed no consistent cross-subject activation patterns (false discovery rate corrected, p<0.05), complicating interpretation within a reductionist framework. This is also suggested by the significant inter-individual differences in feature selection, making it difficult to establish a universal intervention model across participants. Second, the premise of establishing an individualized decoder is the pre-scan before training, potentially necessitating numerous repeated trials, which can be challenging to implement in certain scenarios. For instance, to enhance pilots’ performance in emergencies, it is impractical to artificially induce emergencies multiple times for the purpose of extracting brain patterns associated with their agile responses. Finally, the experimental setup did not employ short-channel regression, which might introduce confounding effects from physiological noise and should be considered in subsequent research.

## 5 Conclusion

Machine learning classifiers successfully decoded conflict states. Three 25-min DecNef sessions enhanced interference control without stimulus exposure, with the effects lasting ≥1 week. This approach enables precise neuromodulation and has translational potential for clinical and cognitive enhancement. Due to the need for some pre-scan trials to train the decoder, a cost-benefit trade-off should be considered in translation.

## Data Availability

The data presented in this study are available on request from the corresponding author.

## References

[1] Scarpina F. Tagini S., “The Stroop color and word test,” Front. Psychol. 8, 557 (2017). 10.3389/fpsyg.2017.00557 28446889 PMC5388755

[2] Yeung M. K. Lee T. L. Chan A. S., “Neurocognitive development of flanker and Stroop interference control: a near-infrared spectroscopy study,” Brain Cognit. 143, 105585 (2020). 10.1016/j.bandc.2020.105585 32535484

[3] Ramot M. Martin A., “Closed-loop neuromodulation for studying spontaneous activity and causality,” Trends Cognit. Sci. 26, 290–299 (2022). 10.1016/j.tics.2022.01.008 35210175 PMC9396631

[r4] Sitaram R. et al., “Closed-loop brain training: the science of neurofeedback,” Nat Rev Neurosci. 18, 86–100 (2017). 10.1038/nrn.2016.164 28003656

[r5] Liu L. Chen Z., “The application of closed-loop brain training: near-infrared spectroscopy (NIRS) neurofeedback,” in Proc. 2nd Int. Symp. on Artif. Intell. for Med. Sci. (ISAIMS '21), Association for Computing Machinery, New York, NY, USA, pp. 263–267 (2021).

[6] Farkhondeh Tale Navi F. et al., “Closed-loop modulation of the self-regulating brain: a review on approaches, emerging paradigms, and experimental designs,” Neuroscience 483, 104–126 (2022). 10.1016/j.neuroscience.2021.12.004 34902494

[7] Vincent T. D. et al., “Conducting decoded neurofeedback studies,” Soc. Cognit. Affect. Neurosci. 16, 838–848 (2020). 10.1093/scan/nsaa063 PMC8343564 32367138

[8] Shibata K. et al., “Toward a comprehensive understanding of the neural mechanisms of decoded neurofeedback,” Neuroimage 188, 539–556 (2019). NEIMEF 1053-8119 10.1016/j.neuroimage.2018.12.022 30572110 PMC6431555

[9] Diamond A., “Executive functions,” Handb. Clin. Neurol. 173, 225–240 (2020). HACNEU 0072-9752 10.1016/B978-0-444-64150-2.00020-4 32958176

[10] Kang W. et al., “Inhibitory control development: a network neuroscience perspective,” Front. Psychol. 13, 651547 (2022). 10.3389/fpsyg.2022.651547 36300046 PMC9588931

[11] Ishii-Takahashi A. et al., “Prefrontal activation during inhibitory control measured by near-infrared spectroscopy for differentiating between autism spectrum disorders and attention deficit hyperactivity disorder in adults,” Neuroimage-Clin. 4, 53–63 (2014). 10.1016/j.nicl.2013.10.002 24298446 PMC3842411

[12] Munakata Y. et al., “A unified framework for inhibitory control,” Trends Cognit. Sci. 15, 453–459 (2011). 10.1016/j.tics.2011.07.011 21889391 PMC3189388

[13] Borsboom D. Cramer A. Kalis A., “Brain disorders? Not really… why network structures block reductionism in psychopathology research,” Behav. Brain Sci. 42, e2 (2018). BBSCDH 0140-525X 10.1017/S0140525X17002266 29361992

[14] Kotsyubinsky A. P. Kotsyubinsky D. A., “Biological reductionism as an obstacle to the advancement of the biopsychosocial concept of mental disorders,” Consort. Psychiatr. (Eng. Ed. Online) 4, 75–84 (2023). 10.17816/CP15476 PMC11009979 38618641

[15] Erika-Florence M. Leech R. Hampshire A., “A functional network perspective on response inhibition and attentional control,” Nat. Commun. 5, 4073 (2014). NCAOBW 2041-1723 10.1038/ncomms5073 24905116 PMC4059922

[16] Hampshire A. Sharp D. J., “Contrasting network and modular perspectives on inhibitory control,” Trends Cognit. Sci. 19, 445–452 (2015). 10.1016/j.tics.2015.06.006 26160027

[17] Long D. L. Prat C. S., “Working memory and Stroop interference: an individual differences investigation,” Mem. Cognit. 30, 294–301 (2002). MYCGAO 0090-502X 10.3758/BF03195290 12035891

[18] Haynes J. D. Rees G., “Decoding mental states from brain activity in humans,” Nat. Rev. Neurosci. 7, 523 (2006). NRNAAN 1471-003X 10.1038/nrn1931 16791142

[19] Emberson L. L. et al., “Decoding the infant mind: multivariate pattern analysis (MVPA) using fNIRS,” Plos One 12, e0172500 (2017). POLNCL 1932-6203 10.1371/journal.pone.0172500 28426802 PMC5398514

[r20] Ghouse A. Candia-Rivera D. Valenza G., “Multivariate pattern analysis of entropy estimates in fast- and slow-wave functional near infrared spectroscopy: a preliminary cognitive stress study,” in 44th Annu. Int. Conf. IEEE Eng. in Med. & Biol. Soc. (EMBC), pp. 373–376 (2022). 10.1109/EMBC48229.2022.9871839 36085980

[r21] Chen T. et al., “Decoding different working memory states during an operation span task from prefrontal fNIRS signals,” Biomed. Opt. Express 12, 3495 (2021). BOEICL 2156-7085 10.1364/BOE.426731 34221675 PMC8221954

[r22] Trambaiolli L. R. et al., “Subject-independent decoding of affective states using functional near-infrared spectroscopy,” Plos One 16, e0244840 (2021). POLNCL 1932-6203 10.1371/journal.pone.0244840 33411817 PMC7790273

[23] Marsicano G. Bertini C. Ronconi L., “Decoding cognition in neurodevelopmental, psychiatric and neurological conditions with multivariate pattern analysis of EEG data,” Neurosci. Biobehav. Rev. 164, 105795 (2024). NBREDE 0149-7634 10.1016/j.neubiorev.2024.105795 38977116

[24] Iordan C. R. et al., “Sculpting new visual categories into the human brain,” Proc. Natl. Acad. Sci. U. S. A. 121, e2410445121 (2024). 10.1073/pnas.2410445121 39625982 PMC11648923

[25] Huppert T. J. et al., “HomER: a review of time-series analysis methods for near-infrared spectroscopy of the brain,” Appl. Opt. 48, D280 (2009). APOPAI 0003-6935 10.1364/AO.48.00D280 19340120 PMC2761652

[26] Tachtsidis I. Scholkmann F., “False positives and false negatives in functional near-infrared spectroscopy: issues, challenges, and the way forward,” Neurophotonics 3, 031405 (2016). 10.1117/1.NPh.3.3.031405 27054143 PMC4791590

[27] Ramot M. et al., “Covert neurofeedback without awareness shapes cortical network spontaneous connectivity,” Proc. Natl. Acad. Sci. U. S. A. 113 E2413–E2420 (2016). 10.1073/pnas.1516857113 27071084 PMC4855583

[28] Shibata K. et al., “Perceptual learning incepted by decoded fMRI neurofeedback without stimulus presentation,” Science. 334, 1413–1415 (2011). 10.1126/science.1212003 22158821 PMC3297423

[29] Koizumi A. et al., “Fear reduction without fear through reinforcement of neural activity that bypasses conscious exposure,” Nat. Hum. Behav. 1, 0006 (2017). 10.1038/s41562-016-0006 PMC5628620 28989977

[30] Faul F. et al., “Statistical power analyses using G*Power 3.1: tests for correlation and regression analyses,” Behav. Res. Methods 41, 1149–1160 (2009). 10.3758/BRM.41.4.1149 19897823

[31] Da Silva J. C. De Souza M. L., “Neurofeedback training for cognitive performance improvement in healthy subjects: a systematic review,” Psychol. Neurosci. 14, 262 (2021). 10.1037/pne0000261

[32] Amano K. et al., “Learning to associate orientation with color in early visual areas by associative decoded fMRI neurofeedback,” Curr. Biol. 26, 1861–1866 (2016). 10.1016/j.cub.2016.05.014 27374335 PMC4961545

[33] Oldfield R. C., “The assessment and analysis of handedness: the Edinburgh inventory,” Neuropsychologia 9, 97–113 (1971). NUPSA6 0028-3932 10.1016/0028-3932(71)90067-4 5146491

[34] Ye J. C. et al., “NIRS-SPM: statistical parametric mapping for near-infrared spectroscopy,” Neuroimage 44, 428–447 (2009). NEIMEF 1053-8119 10.1016/j.neuroimage.2008.08.036 18848897

[35] Carlen M., “What constitutes the prefrontal cortex?,” Science 358, 478 (2017). SCIEAS 0036-8075 10.1126/science.aan8868 29074767

[36] Ashton K. et al., “Time-resolved multivariate pattern analysis of infant EEG data: a practical tutorial,” Dev. Cognit. Neurosci. 54, 101094 (2022). 10.1016/j.dcn.2022.101094 35248819 PMC8897621

[37] Hou X. et al., “NIRS-KIT: a MATLAB toolbox for both resting-state and task fNIRS data analysis,” Neurophotonics 8, 010802 (2021). 10.1117/1.NPh.8.1.010802 33506071 PMC7829673

[38] Pinti P. et al., “Current status and issues regarding pre-processing of fNIRS neuroimaging data: an investigation of diverse signal filtering methods within a general linear model framework,” Front. Hum. Neurosci. 12 (2019). 10.3389/fnhum.2018.00505 PMC6336925 30687038

[39] Chiarelli A. M. et al., “Differential pathlength factor in continuous wave functional near-infrared spectroscopy: reducing hemoglobin’s cross talk in high-density recordings,” Neurophotonics 6, 035005 (2019). 10.1117/1.NPh.6.3.035005 31423455 PMC6689143

[40] Handwerker D. A. Ollinger J. M. D’Esposito M., “Variation of BOLD hemodynamic responses across subjects and brain regions and their effects on statistical analyses,” Neuroimage 21, 1639–1651 (2004). NEIMEF 1053-8119 10.1016/j.neuroimage.2003.11.029 15050587

[41] Domingos P., “A few useful things to know about machine learning,” Commun. ACM. 55, 78–87 (2012). CACMA2 0001-0782 10.1145/2347736.2347755

[42] Hastie T. Tibshirani R. Friedman J., The Elements of Statistical Learning: Data Mining, Inference, and Prediction, Springer (2009).

[43] Lingwei Z. et al., “The emergent property of inhibitory control: implications of intermittent network-based fNIRS neurofeedback training,” Front. Hum. Neurosci. 19, 1513–1527 (2025). 10.3389/fnhum.2025.1513304 PMC11913857 40104768

[44] Kruggel F. Cramon D. Y. V., “Temporal properties of the hemodynamic response in functional MRI,” Hum. Brain Mapp. 8, 259–271 (1999). HBRME7 1065-9471 10.1002/(SICI)1097-0193(1999)8:4<259::AID-HBM9>3.0.CO;2-K 10619419 PMC6873324

[45] Schroeter M. L. et al., “Prefrontal activation due to Stroop interference increases during development—an event-related fNIRS study,” Neuroimage 23, 1317–1325 (2004). NEIMEF 1053-8119 10.1016/j.neuroimage.2004.08.001 15589096

[46] Gagnon L. et al., “Short separation channel location impacts the performance of short channel regression in NIRS,” Neuroimage 59, 2518–2528 (2012). NEIMEF 1053-8119 10.1016/j.neuroimage.2011.08.095 21945793 PMC3254723

[r47] Zhou Q. et al., “Cortical activation and functional connectivity during a verbal fluency task in patients with chronic insomnia: a multi-channel NIRS study,” J. Psychiatr. Res. 179, 270–278 (2024). JPYRA3 0022-3956 10.1016/j.jpsychires.2024.09.025 39332354

[48] Strangman G. et al., “A quantitative comparison of simultaneous BOLD fMRI and NIRS recordings during functional brain activation,” Neuroimage 17, 719–731 (2002). NEIMEF 1053-8119 10.1006/nimg.2002.1227 12377147

[49] Macduffie K. E. et al., “Single session real-time fMRI neurofeedback has a lasting impact on cognitive behavioral therapy strategies,” NeuroImage: Clin. 19, S452034608X (2018). 10.1101/258095 PMC6005804 29922575

[r50] Yamashita A. et al., “Connectivity neurofeedback training can differentially change functional connectivity and cognitive performance,” Cereb. Cortex 27, 4960–4970 (2017). 53OPAV 1047-3211 10.1093/cercor/bhx177 28922830

[51] Cortese A. et al., “Decoded fMRI neurofeedback can induce bidirectional confidence changes within single participants,” Neuroimage 149, 323–337 (2017). NEIMEF 1053-8119 10.1016/j.neuroimage.2017.01.069 28163140 PMC5755700

[52] Huang W. et al., “Neurofeedback training with an electroencephalogram-based brain-computer interface enhances emotion regulation,” IEEE Trans. Affect. Comput. 14, 998–1011 (2021). 10.1109/TAFFC.2021.3134183

